# Mitral valve replacement with small cavity of left ventricle

**DOI:** 10.1186/1749-8090-8-S1-O288

**Published:** 2013-09-11

**Authors:** V Popov, V Lazoryshinets, V Shimon, Yu Lukach

**Affiliations:** 1 Department of Acquired Heart Diseases, Natonal Amosov's Institute of Cardiovascular Surgery, Kyiv, Ukraine

## Background

To determine significance of patient-prosthesis mismatch (PPM) (indexed effective orifice area < 1,2 cm²/m²) after isolated mitral valve replacement(MVR) in pts with small cavity of left ventricle (SCLV) (end-diastolic volume (EDV) ≤ 75 ml) during hospital period.

## Materials

1811 adult patients (pts) with isolated mitral valve disease MVR were operated in Institute from 01.01.2000 till 01.01.2007. There were 127 (7,0%) pts with SCLV. Among them 48(37,8%) males and 79(62,2%) females in average age 53,2+7,1 yy. 110 (86,6%) pts belonged to IV NYHA class of heart failure, 17 (13,4%) – to III. Previous closed mitral comissurotomy was performed in 31 (24,4%) pts, to 7 pts – twice (closed recomissirothomy). Average body surface area (BSA) was 1,87±0,32 m². Following prostheses were implanted: bileaflet (Saint Jude, Carbomedics, On-X, Edwards-Mira) (n=88) and monodisc as Alcarbon`s type (MIKS, LIKS) (n=40). Following prosthesis sizes were used: 23 mm (n=1), 25 mm (n=74), 26 mm (n=3), 27 mm (n=49).

## Results

Hospital mortality (HM) was 5,5% (n=7). It was higher in cases with 27 mm size of implanted prosthesis - 8,2% (n=4/49) than in other group - 3,8% (n=3/78) (p<0,01). PPM were marked in 21 (16,5%) pts with BSA >1,75 m² and size of prothesis 25 mm but there were no influence on HM. Heart failure and PPM were marked in 5 (3,9%) pts with BSA>1,75 m², size of prothesis 25 mm and cavity of LV (EDV≤50 ml). Risk-factors for PPM in SCLV group of pts on hospital stage were: small cavity of LV (EDV≤50 ml) especially in pts with BSA>1,75 m², previous operation, pulmonary hypertension, mitral valve calcification 3+, duration of rheumatic disease ≥25 years.

## Conclusion

Pts with SCLV are in group of higher risk for operation and increasing risk of PPM. In these cases implantation of 25 mm prosthesis is expedient, but for pts with EDV≤50 ml and BSA>1,75 m² it may lead for significant PPM and heart failure. 23 mm prosthesis may use in pts with body mass ≤ 45 kg (BSA<1,5 m²).

